# The Critical Photographic Variables Contributing to Skull-Face Superimposition Methods to Assist Forensic Identification of Skeletons: A Review

**DOI:** 10.3390/jimaging10010017

**Published:** 2024-01-07

**Authors:** Sean S. Healy, Carl N. Stephan

**Affiliations:** Laboratory for Human Craniofacial and Skeletal Identification (HuCS-ID Lab), School of Biomedical Sciences, The University of Queensland, Brisbane 4072, Australia; c.stephan@uq.edu.au

**Keywords:** skull, face, video, photograph, registration, focus distance, superimposition

## Abstract

When an unidentified skeleton is discovered, a video superimposition (VS) of the skull and a facial photograph may be undertaken to assist identification. In the first instance, the method is fundamentally a photographic one, requiring the overlay of two 2D photographic images at transparency for comparison. Presently, mathematical and anatomical techniques used to compare skull/face anatomy dominate superimposition discussions, however, little attention has been paid to the equally fundamental photographic prerequisites that underpin these methods. This predisposes error, as the optical parameters of the two comparison photographs are (presently) rarely matched prior to, or for, comparison. In this paper, we: (1) review the basic but critical photographic prerequisites that apply to VS; (2) propose a replacement for the current anatomy-centric searches for the correct ‘skull pose’ with a photographic-centric camera vantage point search; and (3) demarcate superimposition as a clear two-stage phased procedure that depends first on photographic parameter matching, as a prerequisite to undertaking any anatomical comparison(s).

## 1. Introduction

**Figure 1:**
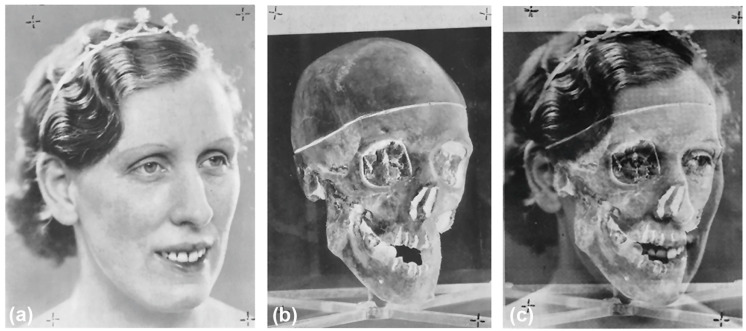
An example of a photographic superimposition of the skull and face for identification purposes [9]. (**a**) AM reference face photograph. (**b**) Skull photograph (greyscale inverted) obtained using next-to-identical photographic parameters to those used for studio portrait photography in (**a**). (**c**) Superimposition of (**a**,**b**) for anatomical evaluation of skull-face alignment. Note that in this case, the teeth are missing from the skull as the perpetrator removed them during dismemberment (in an effort to hinder alternate dental identification processes). Images reproduced from [9] pp. 162–163.

When a skeleton cannot be identified via the usual processes of DNA or dental record comparison, video superimposition (VS) may be used to compare the skull to the relevant antemortem (AM) facial photographs in the hope that new leads assist identification [1,2,3,4] (Figure 1). The VS method is most potent when: (1) the skull is well-preserved; (2) the AM photographs of the potential victim used for comparison present in-focus high-resolution images of the subject; (3) these photographs are acquired just prior to death; (4) these photographs exhibit teeth in an open-mouth smile (especially for frontal view comparisons); and (5) multiple such AM photographs of the head exist in different face views (see, e.g., [5,6,7,8] and Figure 1). 

The VS method depends on the motion-picture overlay of the skull with a still-frame AM face photograph to evaluate the degree of anatomical match between the two images [3,4,10,11,12,13]. This photographic dependence fundamentally sets the method to be a photographic technique, however, input from photographers has been the exception rather than the rule, see, e.g., [14]. Generally, forensic odontologists [4,15,16,17] and anthropologists [7,13,18,19,20,21] have driven method progress, resulting in some important, though basic, photographic and optical parameters being overlooked. The aim of this paper is to bring these photographic considerations to the fore. 

**Figure 2:**
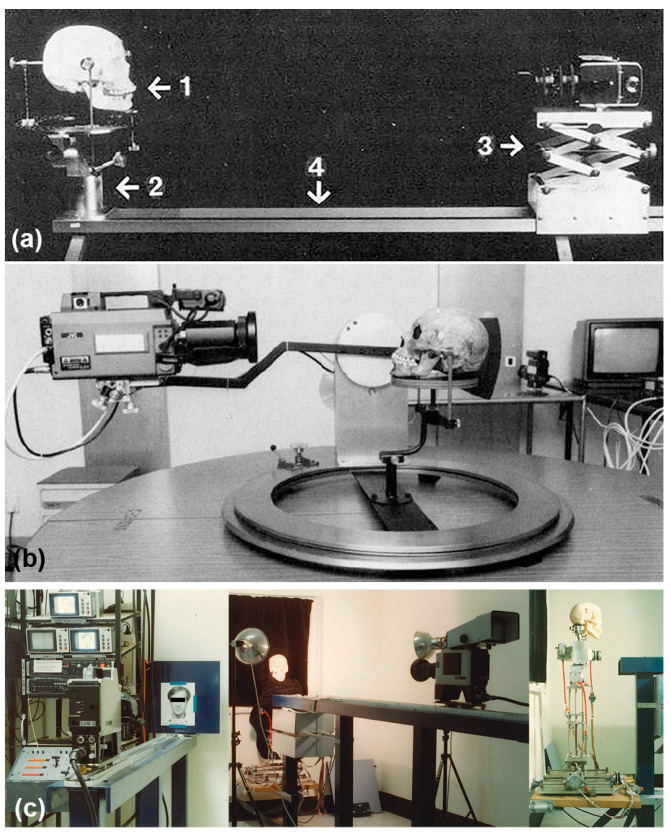
Example superimposition devices. (**a**) Brocklebank and Holmgren’s [16] superimposition gantry. Reproduced from the work presented in [16] p. 1215 with permission from ASTM International. (**b**) McKenna’s [28] superimposition device. Note here that the camera in this system unconventionally moves around a fixed skull—for most VS systems it is the reverse. Reproduced from the work presented in [28] p. 753 with permission from ASTM International. (**c**) Taylor and Brown’s [29] computer-controlled superimposition device that remotely operates the skull position (Five degrees of freedom via electric motors, and one degree (focus distance) by manual movement of the camera) via electric motors. Reproduced from the work presented in [29] with permission from Jane A. Taylor.

Historically, two video cameras have been employed, one recording a hardcopy of the facial photograph and the other recording the skull [4,5,19,21,22,23,24]. A video mixer is used to overlay or superimpose the two images such that they can be directly compared [4,25]. Often the skull is orientated by eye (to match the pose of the head in the AM image), on a simple cork ring (or beanbag) that supports the skull [19,21,22]. In some more advanced superimposition systems, a computer control unit is used to adjust the rotation/pan/tilt actions of the skull via robotic motors (Figure 2) [1,4,26]. More recently, there have been attempts to further computerise the method by comparing 3D acquired skull surface meshes to 2D facial photographs, rather than using the original physical skull [6,27].

Craniofacial superimposition was first employed in a forensic context using still-frame cameras in 1935 [9]. Following the development of magnetic tape video recording in the 1950s, there was a shift to VS in the mid-1970s [11,13,24,25,30,31]. The first VS methods were executed in PAL/NTSC analogue television encoding formats and using video home system (VHS) recording [11,13,25,30,31]. The shift from still- to motion-picture permitted real-time, dynamic, evaluation of skull position and orientation relative to the AM image, which offered the substantial improvements in speed and less tedious image alignments compared to still-frame photography [4]. The downside to this manoeuvre was a major decrease in image resolution from the 35 mm still-frame standard, the equivalent of approximately 20 megapixels (MP), to only 0.3072 MP for NTSC (or 0.4408 MP for PAL [32]). This image resolution loss, by a factor of almost 40× in contrast to still-frame photography, was extremely large, yet its detrimental impact on the accuracy of methods or identity decisions surprisingly goes unaddressed in prior scientific literature. Irrespective of recent attempts to move VS entirely into the digital domain [6,27], the methods continue to fundamentally hinge on photographic principles because the skull (whatever format is used: raw or 3D scan) is always compared to a two-dimensional AM photograph, which serves as the key identity reference.

Since its development, the VS method has been deployed across many continents/countries (Table 1). It has been a popular modality in developing countries as pre-existing facial photographs are common and relatively easy to obtain, in contrast to DNA reference samples or AM dental records/X-rays [4,27,33]. It has additionally been suggested to be effective for disaster victim identification [27] and has been accepted by courts in multiple countries as a valid means of skeletal identification [14,34,35,36]. More recently, however, its utility for scientific identification has increasingly been questioned as accuracy and reliability issues have emerged in parallel (see below). 

Historically, VS and/or photographic superimposition has been employed in some high-profile forensic cases, including dismemberments [9], serial killings [37,74,75], mass burials [76] and the identification of the Nazi war criminal Joseph Mengele [36]. Despite more recent and improved video technology to execute methods [2,10,27], concerns over accuracy have accumulated with findings of poor laboratory-based performances [53,62] and/or erroneous case decisions (later verified through alternate testing avenues using more reliable methods). These erroneous casework examples include both false positives (wrongful identifications; see, e.g., [76]) and false negatives (wrongful exclusions; see, e.g., [74,75]). 

The ongoing issues with reliability have driven increasingly common recommendations in the field that the method only be used as an exclusionary tool [1,10,19,22,27], not for positive identifications. While this exclusionary use is now generally accepted [1,10,19,22,27], it should be noted that this approach provides no safety net for false negatives, which continue to be problematic. That is, unreliable methods, even if reserved for exclusions are still likely to produce erroneous results—a factor that has received far less attention in craniofacial superimposition literature [8,39,77]. Here it should be noted that false negative outcomes can be as unfavourable as false positives in some circumstances, such as serial killing cases, if made early in the victim sequence—for a real-world example see [74,75]. Exclusionary use of unreliable methods may also generate a false sense of utility if it is not tempered by a priori probabilities that positive matches often represent rarer events than negative ones (because there is only ever one person who represents a ground truth correct match). That is, exclusionary use of poorly performing methods to make exclusions are often likely to result in correct exclusions by chance alone, simply because the majority of comparisons in VS concern ground truth negative matches [8].

So far, VS methods have universally been used without a means to scientifically estimate the photographic focus distance—the distance between the camera and the subject used for AM photography [77]. While this has been recognized by some as highly problematic [4,19,59,78], until recently, there has been no good forthcoming solution and many practitioners have continued to use VS despite the inability to estimate the focus distance. This results in the comparison of skull and face images that are not photographically equivalent [4,8,23,59,77,78,79]. This highly problematic situation is openly referred to in best-practice method guidelines, which specify focus distance as an important factor, however, no adequate practical solutions to resolve this factor are provided by the very same guidelines [5]. As the photographic demands for image-to-image comparison in a 1:1 fashion are not being met, e.g., by matching the focus distance of PM photographs to the AM conditions, it is perhaps not surprising that present-day superimposition methods have been found to produce errors in scientific tests [53,62,76]. Redressing the focus distance issue, such that VS methods include the ability to accurately estimate this photographic variable is, therefore, critical.

## 2. The Skull Pose versus Camera Vantage Point Conundrum

**Figure 3:**
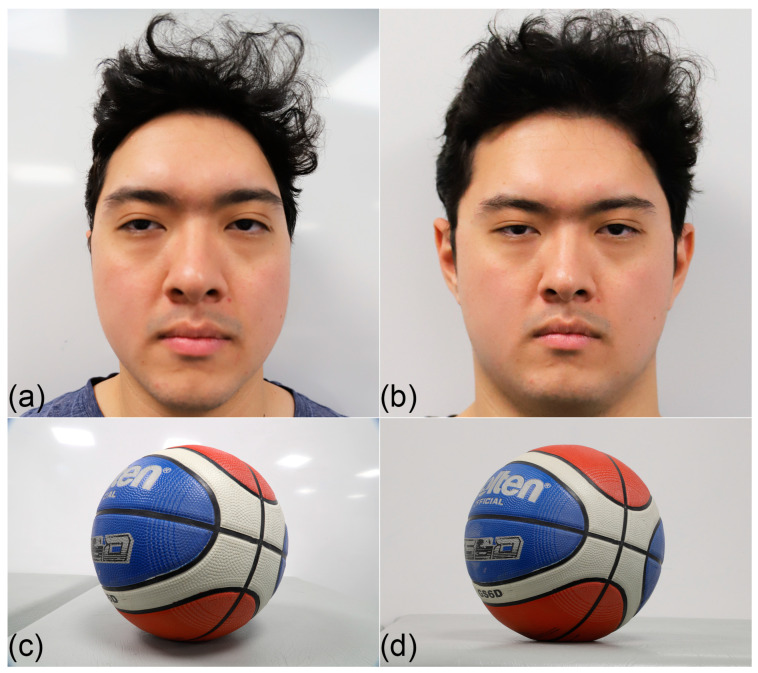
Example of the perspective effects of focus distance on a subject. (**a**) Photograph of the first author (SH) at focus distance of 0.5 m acquired with a Canon^®^ 6D Mark II camera body fitted with a Canon^®^ 24–105 mm zoom lens. (**b**) Photograph of the first author at focus distance of 3 m acquired with the same Canon^®^ 6D Mark II camera body and Canon^®^ 24–105 mm zoom lens as (**a**). Note that the subject in (**a**,**b**) is identical with images taken moments apart, but the recorded anatomical/physical appearance of the face is very different. E.g., the face appears without the ears in (**a**) but not in (**b**) due to the camera recording different peripheral edges of the face. (**c**) Photograph of a basketball at focus distance of 0.3 m acquired with a Canon^®^ 6D Mark II camera body fitted with a Canon^®^ 24–105 mm zoom lens. (**d**) Photograph of the same basketball in the same position as (**c**), but at focus distance of 1.5 m using the same Canon^®^ 6D Mark II camera body and Canon^®^ 24–105 mm zoom lens as (**c**). Note that a different amount of the printed text is visible on the basketball in (**d**) relative to (**c**) and that the focus distance sets the perspective of the subject, not the focal length of the lens [8,80].

In present-day superimposition methods, attention has been awarded to setting the skull’s pose, so it matches the AM reference image [4,47,49,50]. This action effectively reduces the VS problem to one of a mobile skull in front of a fixed camera and is, in part, predisposed by the use of equipment and instruments designed to enable skull movement (5-degrees) rather than camera movement (1-degree; see, e.g., Figure 2c). This is problematic, as mentioned above, as it predisposes the focus distance—a critical factor setting the skull/face perspective (Figure 3)—to be paid little attention or go ignored entirely [23,59,78,80]. 

**Figure 4:**
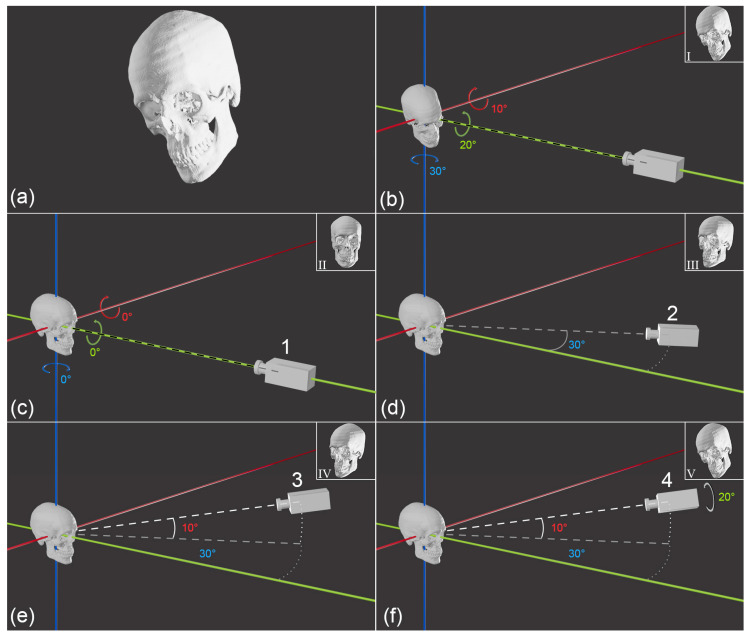
The equivalence of ‘skull pose’ (to a fixed camera) and camera vantage point (in relation to a fixed skull). (**a**) Reference view of the skull targeted by both skull pose and camera vantage point. (**b**) Setting the skull pose in front of a stationary camera and from a starting zero origin to *x*-axis rotation = 10°, *y*-axis rotation = −20°, *z*-axis rotation = −30°. The resultant camera view is shown at inset I. (**c**–**f**) Matching the reference skull view via camera vantage point matching: (**c**) starting zero origin position; (**d**) step 1: camera movement on the x/y plane such that the camera line-of-sight is 20° from the *y*-axis; (**e**) step 2: additional camera movement 10° above *x*/*y*-axis plane; (**f**) step 3: additional camera rotation around line of sight by 20° results in the targeted reference view of the skull: compare inset I in (**b**) to inset V in (**f**). This equivalence can be further visualised in Video S1.

**Figure 5:**
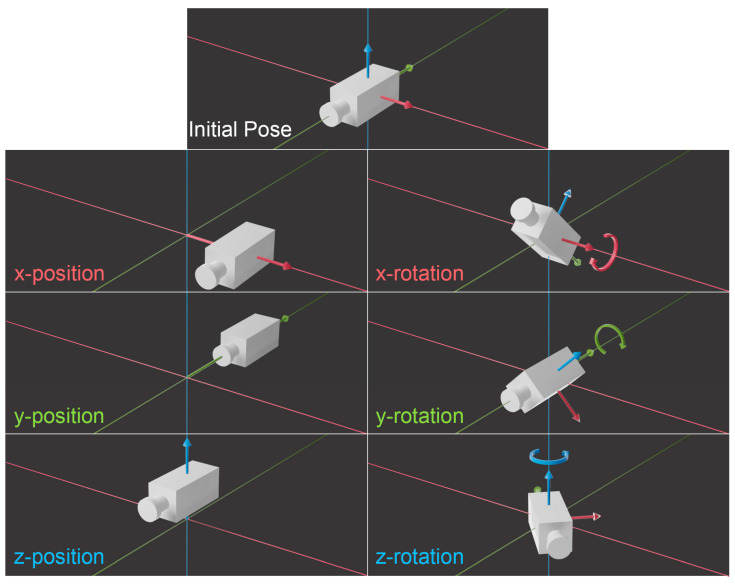
The 6 degrees of freedom of a camera in 3D space. Note here that the combination of x, y and z position control the focus distance parameter, such that focus distance cannot be ignored. The changes in position and rotation can be further visualised in Video S2.

Rather than formulating VS’s challenge as one of matching skull pose between the two images, the problem is better framed as a camera vantage point question, since that more comprehensively includes the relevant photographic parameters. That is, the spatial orientation question of skull/camera relations emphasises a mobile camera, rather than a mobile skull. There is no loss of information with this approach (Figure 4 and Video S1), rather it is the same exact same scenario simply approached from the other side of the equation, and with the added benefit that all 6 degrees of freedom are awarded explicit attention (Figure 5 and Video S2).

It is important to note that a change to a camera vantage point search does not mandate changes to VS skull mounts or redesign of other equipment. Rather, what we are suggesting is a conceptual change to the way the problem of skull pose matching is treated whereby all aspects of camera pose are equivalently considered without discounting any variable, such as the focus distance. It is possible to convert the correct camera vantage point to the equivalent skull pose using established VS equipment, thereby resulting in the accurate focus distance estimate. 

Note here that we define focus distance, at the start of this subsection, as identical to subject-to-camera distance. This is generally appropriate; however, if one chooses to take a more pedantic approach, the argument may be made that focus distance and subject-to-camera distance are not the same, since a face in a photography may not fall precisely at the focal plane and thus, may not be in focus. Thereby, focus distance may not be the same as the subject-to-camera distance. It is important to note that this circumstance is inapplicable to VS because VS prescribes that the face in the facial photograph to be used for comparison must be clear and in-focus to facilitate accurate analysis (see Section 1). Subsequently, the focus distance and subject-to-camera distance are equivalent in VS.

## 3. Why Focus Distance (y-Translation) Is as Important as Other Head Pose Factors

For spherical objects, such as the head or skull, it is well known in photography (but less appreciated in the superimposition domain) that the focus distance has multiple consequences for the object’s representation on the image sensor and these are often described as perspective (see Figure 3). The focus distance sets: (1) the size of the subject in the field-of-view; (2) the relative size of object features closer to the camera relative to other object features further away from the camera; and (3) what parts of the curved surface at the perimeter of the object register as an ‘edge’ on the 2D image [8,23,59] (Figure 5). This variable is no less important than the other camera vantage point settings (Figure 3), rather it holds equivalent criticality. If the skull is photographed using a different focus distance than the face, then the two structures will not precisely align at superimposition. The relationship is such that the methods are more sensitive to differences at shorter focus distances (<3 m) (Figure 3) [23,59,78,80]. As the appearance of the same object changes when viewed at different distances, it is not scientifically legitimate to compare photographs of skulls and faces for anatomical concordance when the focus distance differs between the images—anatomical correspondence cannot be obtained under these conditions. Focus distance estimation provides an improved basis to conduct VS with improved scientific rigor. It is worth mentioning that the image overlay in Figure 1 is as good as it is because unusual circumstances enabled the focus distance to be precisely estimated: the antemortem photograph represented a professional studio portrait where the portrait photographer could be directly consulted for the camera settings and parameters; the exact same camera and lens used for antemortem photography could be used for skull photography; and known objects recorded in the image could be used as scales (e.g. tiara).

When the focus distance is unknown and/or no non-face objects that can be used as a scale exist within the image, the focus distance can be estimated from the palpebral fissure length of the face, where a lens of known focal length is used [77]. This algorithm (termed *PerspectiveX*) is easily adapted to profile photographs, if these are also accompanied by a frontal photograph of the subject [8,81]. The algorithm works at multiple focus distances between 2–10 m, but since resolution of the face decreases with increasing focus distance, distances under 6 m are recommended [39,81]. 

**Figure 6:**
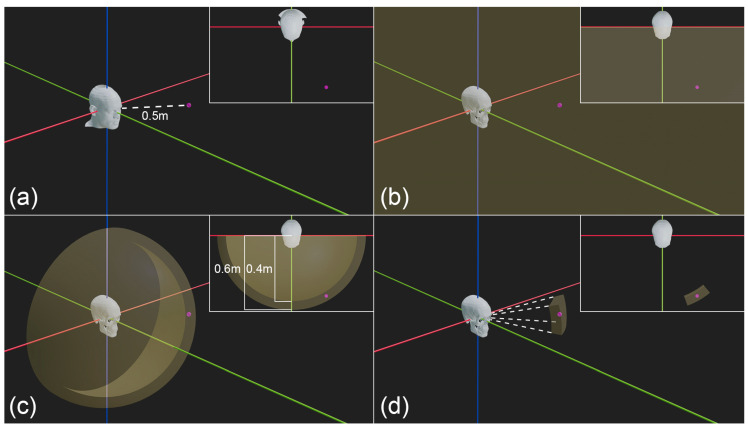
The importance of focus distance for restricting the plausible camera vantage point positions in VS. (**a**) Example ground truth camera position for face photography (pink dot at focus distance of 0.5 m). The red line represents the *x*-axis, the green line the *y* axis and the blue line the *z*-axis.(**b**) Plausible camera vantage point zone, for a frontal view face photograph, without focus distance estimation or constraints by other camera position factors (plausible zone = yellow volume). (**c**) Narrowing of the plausible camera vantage point zone by a focus distance estimate. Note that the yellow sphere is hollow, and a 1% perspective mismatch tolerance has been allowed for the 0.5 m focus distance estimate per [23] providing a focus distance range from 0.4–0.6 m in a spherical pattern around the skull. (**d**) Further narrowing of the plausible camera vantage point zone after additional constraint by the inclusion of other camera position factors and a conservative ±10° rotation error tolerance [83]. Inset for each panel shows superior view of the scene.

For real-world photographs belonging to digital single-lens reflex (DSLR) cameras, *PerspectiveX* typically generates mean signed errors between 3–10% of the ground truth focus distance [8,39,77,81]. This level of error generally translates into less than a 1% change in a chord that holds the same length as Farkas’s sample mean for face height (ground truth cord length = 179 mm [82]; error in photographic representation = 1.8 mm) [77,79]. The error can effectively be halved (0.5×) if a mid-face registration point, such as sellion, is used for skull/face image registration [79]. If the focus distance of an AM image is estimated, the window that represents the plausible zone for matching the camera position is narrowed from all possible positions in front of the skull (180 degree envelope) to only a small range (Figure 6).

Here it should be noted that any AM face photograph with a focus distance of >1 m invalidates the popular use of photographic copy-stands as tools to hold the video cameras, since the focus distance cannot be extended beyond 1 m in these setups to mirror the antemortem photographic conditions (see, e.g., [19,21,22,23]).

## 4. Photographic Equipment Considerations/Factors for Video Superimposition

Like most technology, video has evolved, with modern equipment offering many advantages. For example, VS can nowadays be undertaken on digital flat-panel monitors with less parallax than curved analogue cathode-ray tube (CRT) monitors that were previously common up until the 2000s. Video resolutions are also much higher than those of NTSC/PAL era and are at least as high as 1920 × 1080p (or the equivalent of 2.1 MP). 4K ultra high definition has already arrived as the next standard, providing 8.3 MP resolution. Commercial 8K resolution cameras already exist, and provide 33.2 MP, exceeding the full-frame standard of 20 MP for still-frame photography. These are positive attributes, that favour improved comparisons in VS methods and set new benchmarks for modern-day practice.

### 4.1. Cameras

Due to the constraint of analogue video mixers, VS has traditionally been attained using two video cameras—one dedicated to recording the skull and the other one to recording the 2D photograph of the face [4,6,11,19,21,22,24]. As described in detail below, dual camera requirements are now obsolete, despite still being recommended in VS best practice guidelines as recently as 2015 [5]. Nowadays, output photographic images are digital (or if they exist in print format, they can be converted to digital formats)—removing the requirement for the second video camera to video record a hardcopy still-frame photographic print (see, e.g., Figure 2c).

Further to this, modern DSLR cameras provide (as a stock function) both high-resolution (20 MP+) still-frame images and motion picture acquisition (8.3 MP+) via the very same lens. This streamlined switchable functionality from still-frame to video format on the one camera body enables the video mode to be engaged for real-time dynamic positioning of the skull (just as for traditional VS), prior to a seamless switching to still-frame mode for high-resolution analytical image capture (20 MP). The latest Nikon Z9 DSLR offers even higher resolutions, for example, with 45.3 MP still image and 33.2 MP video [84]. Subsequently, the best of VS (fast and user-friendly alignment) and traditional still-frame superimposition (high resolutions) can now be obtained in modern VS systems using just one DSLR camera. Unlike VS methods of yesteryear, modern-day VS methods thereby blur boundaries between still-frame and motion picture overlay to produce a single unified VS approach. 

### 4.2. Lenses

**Figure 7:**
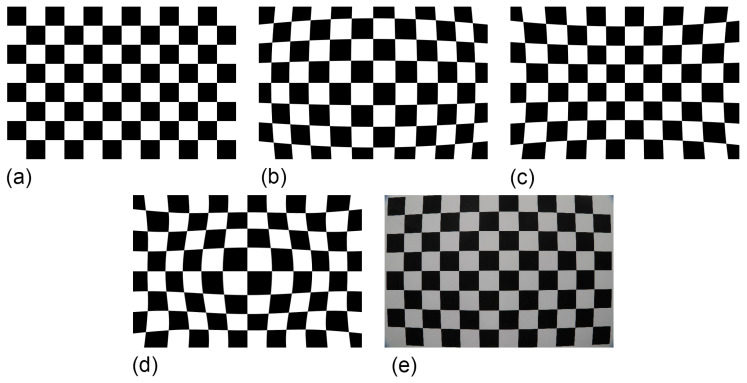
Examples of the different types of radial lens distortions manifested at photography of a regular square grid: (**a**) No distortion; (**b**) Barrel distortion; (**c**) Pincushion distortion; (**d**) Moustache distortion; and (**e**) Real-world barrel distortion from a Canon^®^ 24–105 mm adjustable zoom lens set to 24mm focal length (and as attached to a Canon^®^ 6D Mark II camera body).

Recent studies have shown that the AM photographs selected for VS should be acquired from a prime (fixed focal length) lens [39,77,81], rather than an adjustable or zoom lens, and not from smartphone cameras [81]. As all lenses and cameras possess their own individual idiosyncrasies (something widely recognised in the photography domain, but less so in VS domains), care should be taken that PM images obtained for VS, where possible, come from the same lens as used for AM photography. When this is not possible, pincushion/barrel distortion-free lenses of the same focal length of a different but equivalent lens should be used (Figure 7). 

Whilst all camera lenses possess some level of imperfections, resulting in distortion, the mechanically simpler and less complex fixed prime lenses are widely preferred over adjustable zoom lenses of convenience. Therefore, in addition to seeking DSLR images, investigating authorities responsible for retrieving AM face images for VS should seek: (a) images taken with a high-quality rectilinear, prime lens wherever possible; and (b) centrally located faces within the field-of-view thereby minimizing the impact of lens distortion/aberrations and object stretching across the film plane. When accurate lens correction algorithms exist for the specific given lens model, this distortion can be accounted for, which allows for these photographs to be used. Fish-eye lenses should generally be avoided due their extreme image distortions.

### 4.3. Sensors & Camera Bodies

Although not critical to VS, full-frame sensors are preferred for PM imaging of the skull, and these should hold equivalent or higher resolution to the sensors used for AM face acquisition, such that PM image resolutions do not become a limiting factor. DSLR cameras for PM photography are preferred as these permit user-friendly switching between video and still frame modes with the capacity to acquire very high-resolution still-frame images for VS analysis.

### 4.4. Video-Mixers

Video mixers should be appropriately matched to the specifications of the camera equipment being used for photography (i.e., should not down-sample the camera image resolutions). Ideally, video mixers should be digital devices that pair to a single camera and one that permits seamless switching between still frame and video modes, as described above. (Analogue mixers that require two video inputs to achieve a VS are now rendered obsolete by digital mixers that enable direct input of digital images without a second video camera attachment.) 

### 4.5. Skull Clamps & Positioning Machines

**Figure 8:**
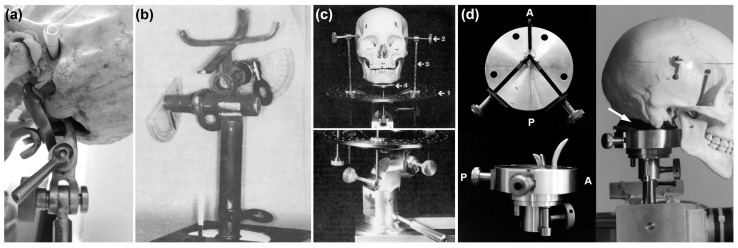
Different types of skull clamps previously used to assist skull positioning in VS and in addition to camera gantries (Figure 2): (**a**) simple craniophore requiring manual adjustment (image acquired at MEPROCS 2014 superimposition workshop, Dundee, Scotland, by C.N.S. with permission by Paul T. Jayaprakash); (**b**) Bastiaan et al. [11] skull clamp with protractors for angle outputs per the manual adjustment (reproduced from [11] p. 1374 with permission from ASTM International); (**c**) Brocklebank and Holmgren’s [16] skull clamp using manual screws and manually adjustable stands (reproduced from [16] p. 1216 and p. 1217 with permission from ASTM International); (**d**) Taylor and Brown’s [4] three-prong skull clamp (manual adjustment) with a methyl methacrylate cold-cure acrylic resin pad (white arrow) to yield unique skull base impression as set on the 3-prong mount and robotically controlled head pose by a remote computer (reproduced from [29] with permission from Jane A. Taylor).

Instruments that permit the systematic, controlled and measured positioning of the skull relative to a fixed camera hold utility and have been multiple in their design [4,11,16,85] (Figure 2 and Figure 8). A key feature of these devices is a skull clamp that affixes the cranium (Figure 8), and which is in turn mounted to some kind of moveable or motorized gantry [4,11,16,85]. Ideally, the skull clamps (see, e.g., Figure 8) and camera mounts should enable exact repositioning for photography, with minimal uncertainty, to facilitate both scientific analysis and courtroom repeatability and reproducibility [4,29]. Ideally, gantries should provide: (1) a means for calibration; (2) position recording via detailed increment step movement; and (3) be accompanied by error and uncertainty results that characterize the instrument’s precision for skull photography. So far in the VS literature, all three of these values have been inadequately addressed [8]. Up to the time of this publication, the lowest step increment reported for VS equipment in the literature is 1-degree [4], but this number comes without accompanying reliability or uncertainty test data, as especially applicable across aggregate degree intervals [8]. It is worth noting here that robotic motor specifications do not suffice for whole of gantry movement precision that must be separately assessed [8].

## 5. The Future

In the future, and beyond focus distance concerns, VS will benefit from the following multiple emergent considerations relevant to camera vantage point:

### 5.1. Fixed-Aspect-Ratio Scaling

Methods to adjust for fixed-aspect-ratio scaling that arise as a function of focus distance estimation error require future attention [78]. With relatively small focus distance error, differences in fixed-aspect-ratio scale can be large and require a systematic means for image adjustment and rescaling [78].

### 5.2. Validated Methods for Determining Camera Angles Relative to the Face

Deriving valid methods for determining camera angles from, and relative to, the face in the AM face photograph is important and has not been finalized despite some initial steps:

#### 5.2.1. Anatomical Methods

Several studies have considered anatomical methods for determining the head tilt, lateral flexion, and lateral rotation using anatomical features [33,42,44,45]. However, none of these methods have been subject to validation tests as to their workbench accuracy. It has been proposed, for example, that forward head tilt is measurable from the vertical distance between the tragus and the exocanthion [49]. A trigonometry method has also been proposed for determining rotation from measurements of each exocanthion from the median line [49], however, practical use in test cases have not yielded successful results [77]. In the computer science domain, it is worth noting that similar feature based, but computational methods, that depend on anatomical landmarks are termed model based methods said to utilise facial keypoints, see, e.g., [83]. 

#### 5.2.2. Computer Vision Methods

Head pose estimation has gained considerable attention in the computer vision domain, but mostly in regard to applications that fall outside VS, such as, monitoring of automotive drivers for their safety and assistance [83]. While many computer vision methods have been developed—including classical methods (e.g., template methods, detector array and manifold embedding), segmentation-based methods, deformable models and Perspective-n-Point—head pose estimation remains challenging for unconstrained environments [83], such as that encountered by VS.

Specifically in the VS domain, Perspective-n-Point (PnP) methods have been incorporated into the latest fully computerized VS methodology [65], however, solutions hold limits and challenges remain because the antemortem face is not accompanied by a real-life 3D scan of the person, the skull is a different structure to the face, and any errors in camera parameters used during the PnP process lead to errors in the end estimation result [67,83]. Ultimately, for the VS context where a single 2D antemortem facial photograph forms the sole, limited, reference material, and is specific to idiosyncrasies of just one individual in a single forensic case, a manually conducted camera vantage point estimation may provide the best casework solution.

An urgent address of these factors is paramount, given established and ongoing recommendations that VS methods continue to be used in forensic casework [6,20] despite current limitations, which risk identification inaccuracies. Only when all of the above camera vantage point factors are adequately resolved will scientifically defensible anatomical comparisons of the skull and the face in VS be possible.

## 6. Conclusions

Craniofacial superimposition is often described in the scientific literature as a scientifically legitimate, mature and straightforward undertaking in forensic odontology [4,15,16,17] or anthropology [7,13,18,19,20,21]. However, the method hinges on a number of critical photographic parameters that have not previously been awarded adequate attention and that in practice, make the method both technically challenging and finicky to implement [8,79]. In VS, these photographic parameters must be adequately met if the method is to be technically sound and successful at the downstream anatomical comparison stages. To accomplish this, we suggest anatomy-centric ‘skull pose’ search [4,47,49,50] be abandoned in favour of searches for the appropriate camera vantage point. Only when all six AM camera pose variables in 3D space have been matched (resulting in a correct focus distance) will there be any hope for skull and face comparisons that are photographically (and anatomically) valid. New focus distance estimation capabilities, combined with new technological advancements in photography equipment, including seamless switching between video and still frame recording capabilities, offer renewed hope to establish scientifically verified methods at sufficient resolutions that support detailed anatomical examinations and reliable identity decisions.

## Figures and Tables

**Table 1:** Casework use of superimposition methods by alpha sequence of country and including other major research contributions by publication.

Country	Earliest Recorded Year of Superimposition Use/Research	Earliest Recorded Year of VS Use/Research	Publication Source
Australia	1977	1977	[4,8,11,30,37,38,39]
Canada	1955	Not Specified	[40]
China	1983	1983	[16,41,42]
Czech Republic	2007	2007	[43]
Germany	1976	1976	[24,31,36,44,45]
India	1960	Not Specified	[40,46,47,48,49,50]
Italy	1986	1986	[51,52,53]
Japan	1988	1988	[26,54,55,56,57]
Malaysia	Not Specified	Not Specified	[33,58]
Netherlands	1989	1989	[59]
New Zealand	1981	1981	[60]
Singapore	1989	1989	[61]
South Africa	1953	1986	[35,53,62]
Spain	2007	2007	[63,64,65,66,67,68]
Sri Lanka	1947	Not Specified	[69]
Sweden	1975	Not Specified	[70]
Switzerland	1987	1987	[25]
UK	1935	Not Specified	[9,14,27]
Uruguay	1995	1995	[71]
USA	1971	1976	[10,13,17,19,20,21,22,72,73]

## Data Availability

Not applicable.

## Supplementary Materials

The following supporting information can be downloaded at: https://www.mdpi.com/article/10.3390/jimaging10010017/s1, Video S1: Equivalence of skull pose and camera vantage point; Video S2: 6 degrees of freedom of a camera in 3D space.
